# *Plasmodium falciparum* Spatial Analysis, Western Kenya Highlands

**DOI:** 10.3201/eid1110.050106

**Published:** 2005-10

**Authors:** Otsyula G. Munyekenye, Andrew K. Githeko, Guofa Zhou, Emmanuel Mushinzimana, Noboru Minakawa, Guiyun Yan

**Affiliations:** * Kenya Medical Research Institute, Kisumu, Kenya;; † State University of New York, Buffalo, New York, USA

**Keywords:** Malaria, highland, prevalence, age group, parasite density, gametocyte, K-function, local cluster, research

## Abstract

We carried out a population-based study to determine the unbiased, age-specific *Plasmodium falciparum* prevalence, asexual and sexual parasite density, and spatial distribution to establish rates of infection at a site in western Kenya. Three cross-sectional surveys were carried out in western Kenya highlands. Blood samples were taken from 1,388 persons from 6 months to 75 years of age. Parasite prevalence and densities in the population decreased with age and distance from valley bottoms. Children from 1 to 4 years of age had the highest parasite prevalence (38.8%–62.8%); in adults, prevalence declined to 2.9%–24.1%. Malaria prevalence declined by an average of 19% from July to December 2002 across age groups. These observations suggest that parasite transmission is intense at this altitude. Asexual parasite density indicated clustering near major vector breeding habitats. Variability in seasonal prevalence indicates transmission instability and susceptibility to epidemics.

## Abstract 2

Parasite transmission is intense in the highlands, and these areas are vulnerable to epidemics.

The reemergence of epidemic malaria in the East African highlands (elevation >1,500 m above sea level) is a public health problem (*1*). Research indicates that the mechanisms leading to epidemic malaria in the highlands are complex and are probably due to the concerted effects of factors such as topography (*2*), hydrology (*3*), climate variability (*4*), land-use/land-cover change (*5*), and drug resistance (*6*). Effective disease control calls for a clear understanding of the interaction between these epidemiologic factors.

Perennial malaria transmission in the lowlands has been attributed to high vector densities throughout the year (*7*). Inhabitants of the basin region of Lake Victoria, western Kenya, experienced up to 300 infective bites per year (*8**,**9*). Vector density and transmission intensity in the highlands are much lower than in the lowlands. For example, a transect study from lowland (300 m elevation) to highland (1,700 m elevation) in the Usambara Mountains in Tanzania found a >1,000-fold reduction in transmission intensity between the holoendemic lowland and the hypoendemic highland plateau (*7*). At high altitudes in the highlands and on hilltops, where malaria transmission intensity is low, human populations have poorly developed immunity to malaria because exposures are infrequent (*10*), and persons are vulnerable to severe clinical illness and complications from *Plasmodium* infection (*11**,**12*). High risk for severe malaria is seen in persons living in areas with low-to-moderate transmission intensities (*13*). In such areas, the proportion of asymptomatic persons is usually lower than in high-transmission areas, where *P. falciparum* prevalence and parasite density varies little between seasons (*14*).

Although malaria incidence has been increasing in the East African highlands (*15*), the extent and distribution of malaria infections in the asymptomatic human population are largely unknown. Data on malaria in the western Kenya highlands are derived from hospital clinical records (*16**,**17*) and do not provide a population-based, epidemiologic profile or information on the unbiased prevalence of malaria in local highland populations. Bias in hospital data can arise from poverty, self-medication, and lack of access to hospitals. Detailed parasitologic data are also not normally available from hospital clinical records. Because data on malaria prevalence in local residents are unavailable, we do not know whether whole populations are susceptible to malaria or the risk is skewed toward children, as is seen in malaria-holoendemic regions. In this study, we investigated malaria parasitologic profiles in a population living in a highland zone in the Kakamega district, western Kenya, where epidemics have been reported (*4*) to determine age-specific parasitemia prevalence, age-specific parasite densities, and the spatial distribution of infections.

## Materials and Methods

### Study Site

**Figure 1:**
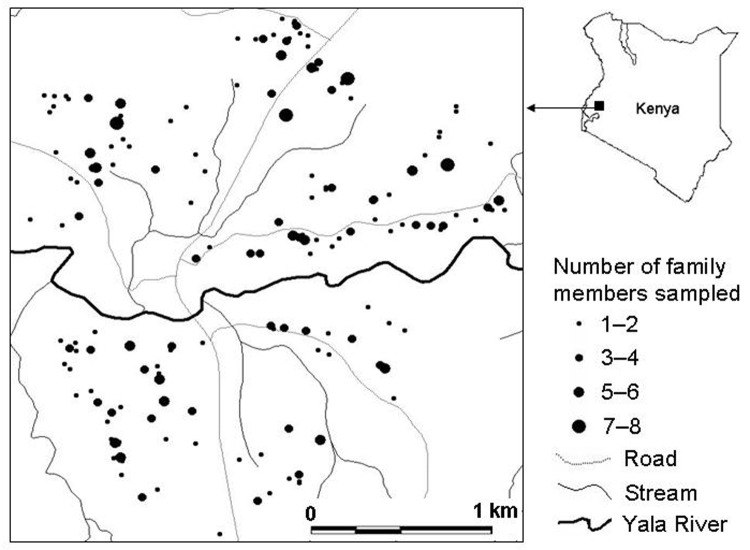
Distribution of residents sampled for *Plasmodium* prevalence, Iguhu village, Kakamega district, western Kenya.

The study site is located in Iguhu village (0°17´N, 34°74´E, elevation 1,450–1,580 m above sea level), Kakamega district, western Kenya, with population ≈11,000. This area experiences 2 rainy seasons and averages 2,000 mm rainfall per year. The long rainy season usually occurs between April and May, with an average monthly rainfall 150–260 mm, while the short rainy season usually occurs between September and October, with an average monthly rainfall 165 mm. Malaria prevalence peaks usually lag 1–2 months after the rain. The mean annual daily temperature is 20.8°C. The area has experienced extensive deforestation and swamp reclamation in recent years as a result of rapid human population growth and the demand for settlement and agricultural land; therefore, only patches of forest remain. Malaria vectors in the area are *Anopheles gambiae* sensu stricto and *A. funestus* (*4**,**18*). Maize is the principal subsistence crop, although vegetables are grown on small, irrigated plots in valley bottoms. The area is bisected by the Yala River (Figure 1); most mosquito larval habitats were found on river banks in the bottom of the valley and on the banks of streams during both dry and rainy seasons (*18*).

### Study Design

The working hypothesis was that no difference would be seen in the prevalence among various age groups and that infections would be randomly distributed. The binomial model was used to estimate the confidence interval (CI). Because the prevalence of malaria was not well known in the area, a 50% estimate, which gives the best sample size, was used for the peak-transmission season, and a 25% estimate was used for the low-transmission season. We calculated the 2 sample sizes with a 95% CI and precision level of 5%:



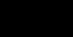



In this equation, *n* is the sample size, *z* is the critical value of the standard normal distribution at the 5% level (1.96), *p* is the malaria prevalence estimate, *q* = 1 – *p*, and *d* is the precision level. The population size was estimated to be 3,000. The sample size obtained was 341 persons for the peak-transmission season and 263 for the low-transmission season.

### Study Population and Data Collection

The study, which involved 1,388 participants, was carried out in 3 cross-sectional surveys undertaken in July 2002 (n = 396), December 2002 (n = 283), and June 2003 (n = 709). Peak malaria transmission occurs 1–2 months after the rainy season; thus, surveys were conducted 1 month after the rains. The short rains before December are usually not adequate to increase transmission, and this period is considered entomologically dry. Figure 1 shows the spatial distribution of residents examined for *Plasmodium* infections in July 2002. The sample size for each age group during each sampling period is shown in the Table. Adults (>18 years of age) were recruited into the study upon giving an informed consent; consent for children (<18 years) was provided by the participants and their parents or guardians. Scientific and ethical clearance was given by Kenya Medical Research Institute and State University of New York at Buffalo ethical review boards. Inclusion criteria were provision of informed consent, age >6 months at recruitment, and no reported chronic or acute illness except malaria. Blood samples were collected by the standard finger-prick method, and thick and thin smears were prepared on labeled slides. The thin and thick blood smears were air dried. The thin smears were fixed in methanol and stained in 4% Giemsa for 30 min with the thick smear. Two experienced technicians examined the slides under ×1,000 oil immersion to identify and count the parasite species. Random checks were carried out on the slide counts by independent microscopists to ensure quality control. Parasite density was scored against 200 leukocytes when the slide was positive; otherwise, the whole slide was carefully scanned before being declared negative. Parasite densities were converted to number of parasites per microliter of blood, assuming a leukocyte count of 8,000 cells/μL (19).

**Table:** Number of samples by age group and sampling occasion

Age group (y)	July 2002	December 2002	June 2003
1	19	9	12
1–4	35	54	79
5–9	128	67	230
10–14	129	54	243
15–19	57	31	32
>19	28	68	113
Total	396	283	709

We examined the monthly malaria infection dynamics in primary school children, age 6–14, in the study area from June 2002 to June 2003, with the microscopic test. Daily rainfall data were collected by using automated weather stations (HOBO, Onset Computer Corporation, Bourne, MA, USA). These data were offloaded monthly from the weather stations.

### Data Analysis

Most of the homes of the participants in the prevalence survey were georeferenced by using a global positioning system (GPS) unit. A database was created with participants' names, ages, GPS location, and parasite density. Participants' names were coded for confidentiality. The relationship between *P. falciparum* prevalence and parasite density was determined across age, season, and distance to the nearest stream/valley. To examine the effects of age on parasite prevalence and infection density, ages were stratified into 6 age groups: <1, 1–4, 5–9, 10–14, 15–19, and >19 years (*20*). Age-specific prevalence was determined by expressing positive blood smears as a percentage of all examined blood smears; only positive slides were considered when the geometric mean parasite density was calculated for each age category. The χ^2^ test was used to determine differences in prevalence among age groups for each survey. Analysis of variance (ANOVA) with logarithm-transformed parasite density was used to examine the difference among age groups and among surveys in parasite density. Nonspatial statistical analyses were conducted by using the JMP statistical package (SAS Institute Inc, Cary, NC, USA).

To determine the spatial distribution pattern of malaria-infected residents, ArcView 3.3 (Environmental Systems Research Institute, Redlands, CA, USA) was used to create spatial-distribution maps of infected and uninfected participants for the 3 surveys. *P. falciparum* infections were tested for clustering by household with the global spatial statistic, the K function, weighted by parasite density (*21**–**23*) by using Point Pattern Analysis software (San Diego State University, San Diego, CA, USA). The global weighted K function, *L*(*d*), examines the spatial dependence of malaria infection by household over a wide range of scales of pattern (*21*). The observed *L*(*d*) function values were tested against the null hypothesis that the spatial distribution of all infected residents in the study area was random, by using 1,000 Monte Carlo iterations. Because children and adults showed significantly different prevalence and parasite density, the global spatial cluster analysis was conducted separately for the 0- to 9-year age group, the 10- to 19-year age group, and adults (>19 years old). The local spatial statistic Gi*(*d*) (*24*) was computed to assess clustering of high parasite densities near a particular transmission source, the Yala River, where most vector breeding sites were found (*18*), for age groups 0–9 and 10–19 years, but not for adults because sample sizes were too small. To correct for multiple comparisons when using Gi*(*d*), significance levels were determined by using Table 3 in reference (*25*).

## Results

### *Plasmodium* Prevalence and *P. falciparum* Density

In addition to *P. falciparum*, we found *P. malariae* and *P. ovale* in our study populations. *P. falciparum* constituted 97.1%, 94.9%, and 94.6% of malaria cases in July and December 2002, and June 2003 surveys, respectively. *P. malariae* constituted 2.9%, 5.1%, and 4.6% of malaria cases, while *P. ovale* was observed only in June 2003 (0.8%). *P. malariae* was often seen in mixed infection with *P. falciparum*. For example, 83.3% of *P. malariae* infections in July 2002 occurred with *P. falciparum*, while 50% of *P. malariae* infections in December 2002, and 81.8% of *P. malariae* infections in June 2003, were accompanied by *P. falciparum*.

**Figure 2:**
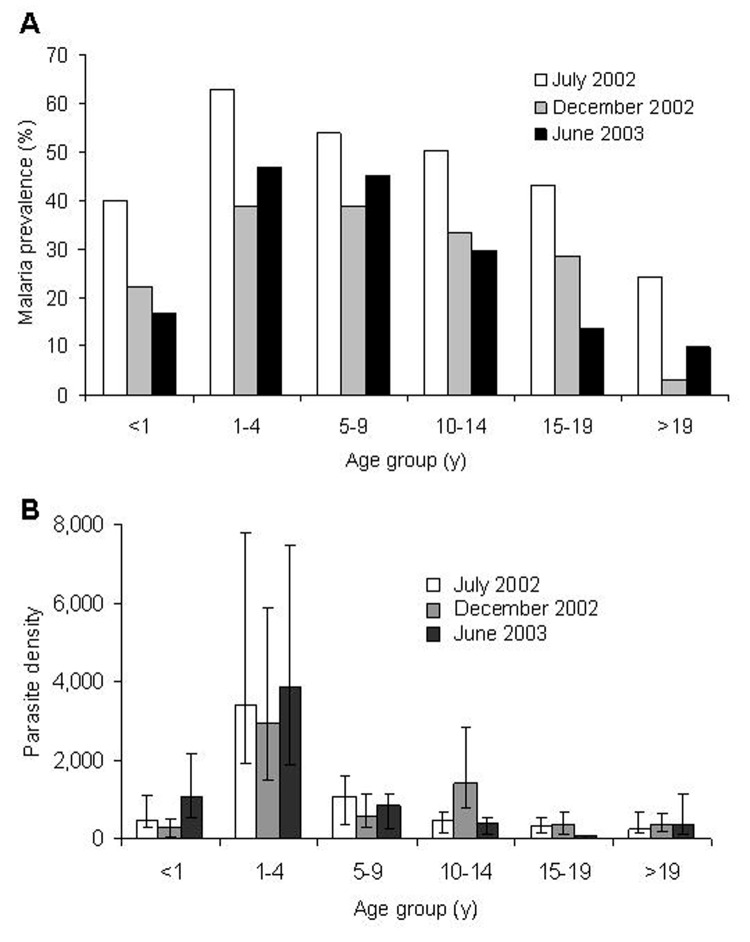
A) *Plasmodium falciparum* trophozoite prevalence. B) Geometric mean of *P. falciparum*–infected erythrocytes per microliter of blood (error bars represent 95% confidence intervals), Iguhu village, Kakamega district, western Kenya.

*P. falciparum* prevalence varied significantly among age groups (homogeneity χ^2^ = 95.82, df = 5, p<0.001) and sampling months (homogeneity χ^2^ = 46.81, df = 2, p<0.001; Figure 2A). Malaria prevalence for all age groups ranged from 50.1% in July 2002 to 27.1% in December 2002. Children 1–9 years of age consistently had the highest prevalence (average 47.0%) in the 3 cross-sectional surveys, while adults >19 years of age showed the lowest prevalence (average 9.5%). The mean parasite density did not vary significantly among survey months (ANOVA, F = 0.53, df = 2, 483, p = 0.59) but did vary significantly among age groups (F = 21.17, df = 5, 483, p<0.001; Figure 2B). For example, children 1–4 years of age had the highest parasite density (geometric mean of 3,469.7 infected erythrocytes/μL blood, 95% CI 2,328.0–5,171.3). This amount was >7-fold higher than the average parasite density among all other age groups (477.8 infected erythrocytes/μL, 95% CI 265.6–715.1; Tukey-Kramer HSD [honestly significant difference] test, p<0.001; Figure 2B).

### *P. falciparum* Gametocyte Prevalence and Density

**Figure 3:**
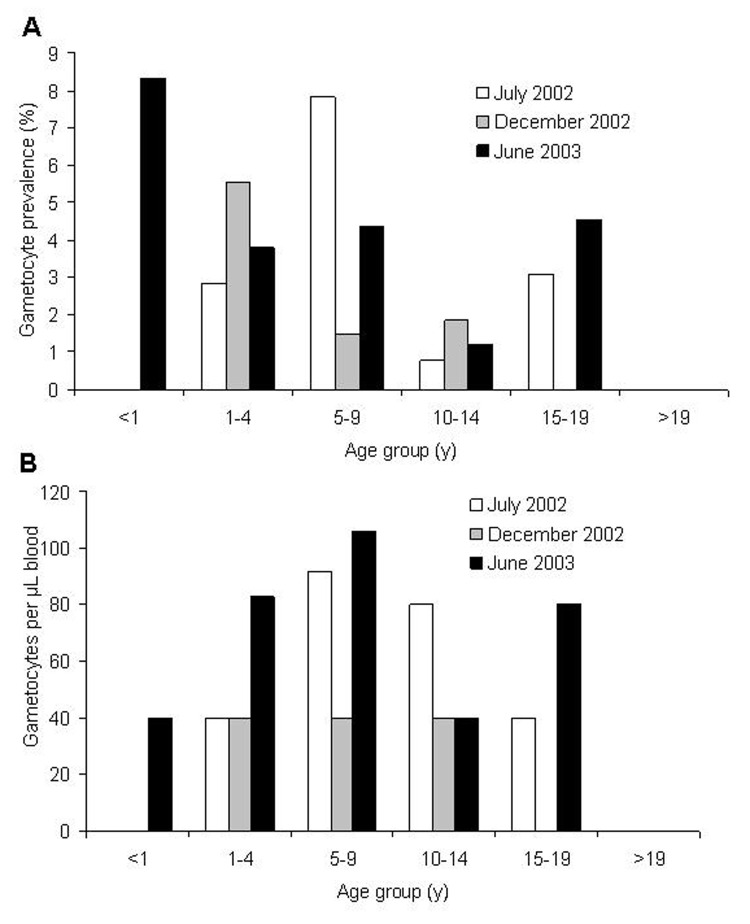
A) *Plasmodium falciparum* gametocyte prevalence. B) Infection densities in different age groups, Iguhu village, Kakamega district, western Kenya.

Overall, *P. falciparum* gametocyte prevalence did not differ significantly among the 3 surveys (homogeneity χ^2^ = 1.34, df = 2, p = 0.51) or age groups (homogeneity χ^2^ = 9.48, df = 5, p = 0.09; Figure 3A). The mean gametocyte prevalence was 2.7% (range 0%–8.3%; Figure 3A). Similarly, gametocyte density did not vary significantly among sampling occasions (ANOVA, F = 0.71, df = 2, 31, p = 0.50) or age groups (F = 0.84, df = 4, 31, P = 0.51; Figure 3B). The geometric mean gametocyte density was 71.7 gametocytes/μL (95% CI 53.8–95.6).

**Figure 4:**
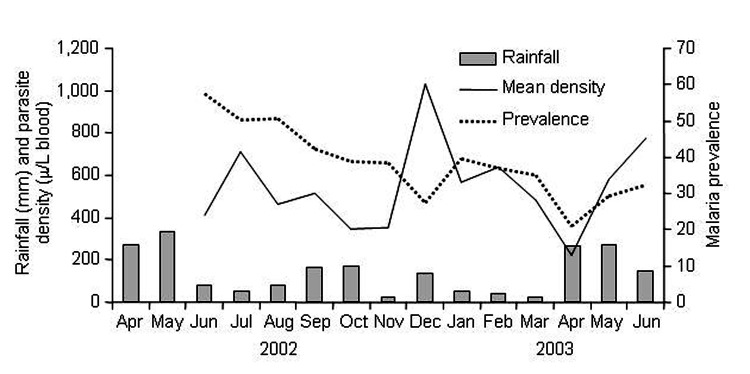
Dynamics of monthly rainfall, monthly (geometric) mean parasite density, and monthly *Plasmodium falciparum* prevalence.

During the study period (June 2002 to June 2003), malaria prevalence showed a general decreasing trend (Figure 4). However, the geometric mean parasite density varied among months. The months with peak parasite density appeared to be 1–2 months behind rainfall peaks.

### Spatial Distribution of Parasite Density

**Figure 5:**
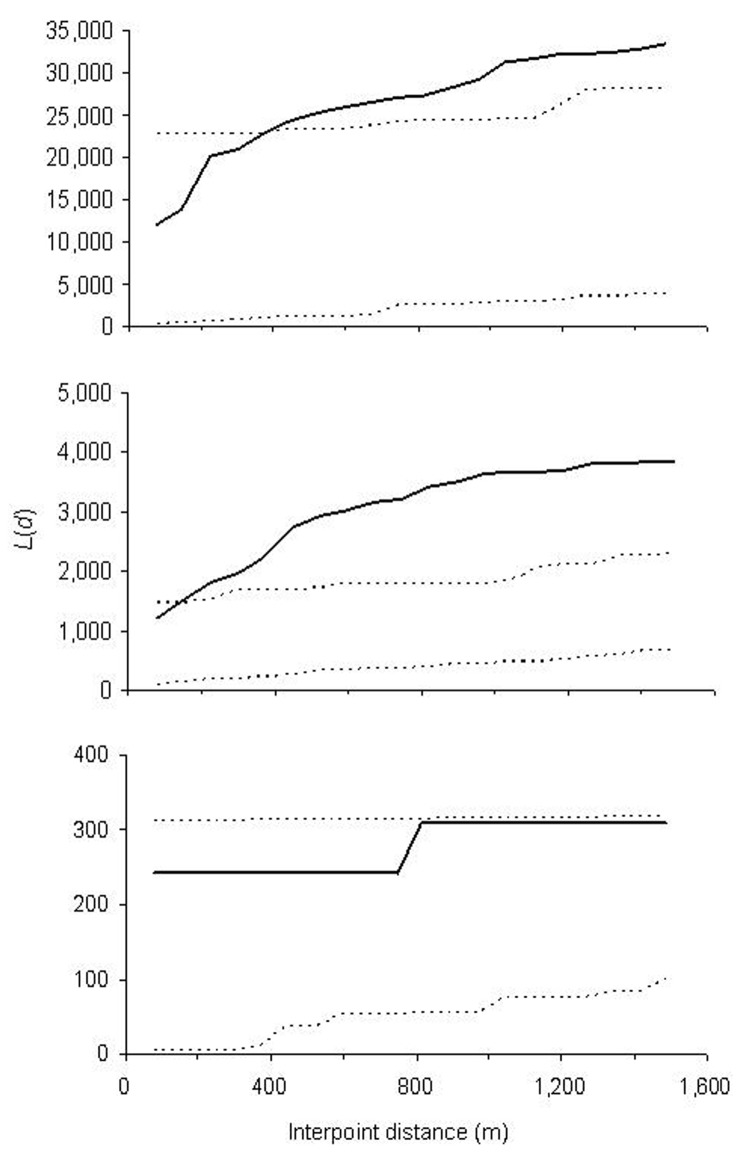
Results of weighted K function analysis on the global spatial clustering of *Plasmodium falciparum* infection intensities in Iguhu for age groups A) 0–9 years, B) 10–19 years, and C) >19 years in the July 2002 survey. The solid line is the observed value of the test statistic *L*(*d*) at a given distance *d*, and dashed lines indicate 95% confidence intervals.

The global weighted K function, *L*(*d*), was used to examine the spatial dependence of malaria infection by household over an interpoint distance of 100–1,400 m for each of the 3 age groups (0.6–9, 10–19, and >19 years). Figure 5 shows measures of the observed *L*(*d*) and the 95% CI plotted for various values of interpoint distance for the July 2002 survey. The spatial distribution of infections is considered evenly dispersed if the observed K function values are below the lower limit of the 95% CI, clustered if above the upper limit, or random if within the 95% CI. The weighted K function indicated that the parasite density distribution pattern was significantly different than expected under complete spatial randomness for age groups 0.6–9 (Figure 5A) and 10–19 years (Figure 5B), but was random in adults (Figure 5C). Spatial clustering for age groups 0.6–9 and 10–19 years occurred at an interpoint distance >150 m. This pattern was consistent with 2 other surveys done in December 2002 and June 2003 (data not shown).

**Figure 6:**
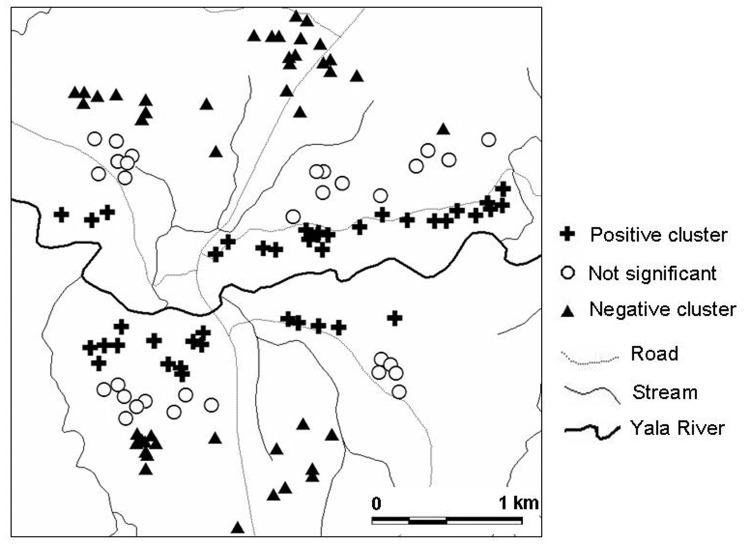
Significant clustering of *Plasmodium falciparum* infection intensities in Iguhu village for children 0–9 years of age.

Local spatial clustering analysis was performed on parasite densities to determine whether higher infection densities were clustered near the Yala River, where most vector breeding sites existed. Figure 6 shows an example of local spatial clustering for age group 0.6–9 years for the July 2002 survey. We found that parasite densities were positively clustered for children living <500 m from the Yala River and negatively clustered for children residing >1,000 m from the river. That is, children living near the river had significantly higher parasite densities than would be expected in random distribution, whereas those farther away from the river exhibited significantly lower parasite densities. Clustering of parasite densities in age group 10–19 years showed a similar pattern, but fewer households had either positive or negative clustering. Similar results were found for the December 2002 and June 2003 surveys (data not shown). Therefore, the patterns of *P. falciparum* parasite density clustering, as shown in Figures 5 and 6, suggest a relationship between a house's distance from the Yala River and clustering of high parasite densities.

## Discussion

In this study, we found that children 1–4 years of age had a 7-fold greater number of parasites in their blood as compared to persons >5 years of age, irrespective of season. The rapid fall in parasite density as age increases suggests age-dependent immunity to *P. falciparum* parasites. The low prevalence of infections among adults further supports this notion. However, the highland topography affects drainage and the distribution of mosquito breeding habitats and therefore also affects exposure to infections. For example, 90% of adult mosquitoes in the study area were collected <300 m from valley bottoms (*26*). Nonuniform exposure patterns to infections as a result of topography and hydrology would lead to similar spatial immunologic profiles. Thus, within the highland human populations, varying states of transmission stability are found.

John et al. carried out an epidemiologic study at an altitude of 2,134 m in the Uasin Gishu district of western Kenya (*27*). They found no difference in reinfection rates between children and adults, which indicates that malaria transmission could be unstable at this altitude. Whether infections translated into clinical disease at the same rate in children and adults is not clear (*28*). Hay et al. (*17*) reported that approximately two thirds of hospital admissions in the Kisii district (elevation 1,500–1,800 m) in the western Kenya highlands were children <15 years of age. Our population-based malaria prevalence data from a village in the Kakamega district (elevation 1,450–1,580 m) indicate that children (1–4 years of age) have the highest malaria prevalence and parasite density, a condition similar to that seen in the holoendemic lowlands. Few records exist of detailed population-based parasitologic and clinical studies of malaria in the highlands of western Kenya, which is why classifying the stability of transmission is difficult. Shililu et al. (*3*) reported prevalence rates in asymptomatic children from 44% in the dry season to 55.4% in the wet season in Mumias, Kakamega district (≈1,500 m above sea level), western Kenya. During the 1990 malaria epidemic in the Uasin Gishu district in western Kenya (elevation 2,000 m), Some (*29*) reported a prevalence of 72% in the general population. In the same year, Ayisi et al. (*30*) observed a mean prevalence of 39.2% in the general population at the Belgut division of the Kericho district (elevation 1,800 m). Our data fall within the range observed by other investigators at similar altitudes. However, we also observed a significant interannual and seasonal variability in malaria prevalence at this highland site. The variation in parasite prevalence between the wet and dry seasons could be explained by differences in vector abundance. For example, the mean monthly anopheline vector abundances increased by 6- to 8-fold in the long rainy season compared to the dry season (*26*).

The prevalence of *P. falciparum* malaria in school children in the low-altitude region of Lake Victoria basin (elevation ≈1,200 m) adjacent to the highlands reaches >80% (31–34), which is much higher than malaria prevalence in the highlands. Therefore, in the highlands, a high proportion of residents was susceptible to infection and clinical disease. Under conditions of hypertransmission, outbreaks and epidemics would result because medical facilities would be unable to cope with the number of persons affected. Malaria instability in this area is related to the proportion of susceptible persons and the variability in transmission. Children 1–4 years of age had the highest parasite densities in their blood; after 5 years of age, densities dropped dramatically. The low prevalence of infections in adults suggests that these persons have developed some degree of immunity as a result of lifelong exposure.

The mean parasite density observed in the 1- to 4-years age group at Iguhu was similar to that observed in the <1-year age group in the lowlands (*32*), which suggests immunity develops more slowly in the highlands. Still, older persons had 7-fold lower parasite loads, which indicates functional, age-dependent immunity.

The age-specific profiles of gametocyte prevalence in the highland site were much lower than those observed in the lowlands; e.g., infants (1–9 months) had a prevalence of 86% in the lowlands, while in the highlands prevalence in this group was 2.9%. Children 5–9 years of age had the highest rate of infection with gametocytes (4.9%) at Iguhu, which is comparatively lower than that reported in the lowland (40%) for the same age group (*32*). The reservoir of malaria infections in the highlands is much lower than in the lowlands. For example, Ayisi et al. (*30*) reported a mean gametocyte prevalence of 1.8% in the Kericho district in the western Kenya highlands, while we observed a mean gametocyte rate of 2.8% in the Kakamega district. By contrast, Githeko et al. (*32*) reported a gametocyte prevalence of 39.1% during the rainy season in the Kisumu district and a rate of 10.8% in the dry season in the Suba district (*35*). Similar to observations made in the lowlands (*32*), the mean gametocyte density in highland residents did not vary significantly among age groups. Compared to what was seen in the lowlands, the proportion of persons carrying gametocytes (the infectious reservoir) in our highland site was 5- to 100-fold less, which suggests a weak transmission system in the highlands.

Spatial analysis of asexual parasite densities indicated clustering in relation to distance from major larval breeding habitats and in relation to age. This phenomenon was not observed in persons living >1,000 m from major breeding sites, which suggests that the rate of parasite transmission may be higher closer to major breeding habitats. This finding is consistent with the spatial distribution of indoor resting vector densities in the area. Our results are consistent with the fact that the risk of malaria is strongly associated with distance from breeding sites (*36**,**37*).

This cross-sectional study in a highland site where malaria epidemics have been reported in the past shows that transmission is intense, particularly after the rainy season, but variable with regard to season and distance from major mosquito breeding habitats. The risk of infection is highly variable within the site, and subsequently, the stability of transmission may reflect this variability. A large host population is available for infection before periods of hypertransmission. Although the population has a functional age-dependent immunity to malaria, as indicated by parasite densities, its development is slower than that found in the holoendemic lowlands, which suggests that this population may be more susceptible to malaria infections and more prone to epidemics compared to the lowland populations.
